# Feasibility, Reproducibility, and Clinical Validity of a Quantitative Chest X-Ray Assessment for COVID-19

**DOI:** 10.4269/ajtmh.20-0535

**Published:** 2020-07-02

**Authors:** Marcello A. Orsi, Giancarlo Oliva, Tahereh Toluian, Carlo Valenti Pittino, Marta Panzeri, Michaela Cellina

**Affiliations:** 1 Department of Radiology, ASST Fatebenefratelli Sacco, Milan, Italy;; 2 Post-graduate School in Radiodiagnostics, Università degli Studi di Milano, Milan, Italy;; 3 Department of Radiology, IRCCS Ospedale San Raffaele, Milan, Italy

## Abstract

Chest X-ray (CXR) is an essential first-line tool in COVID-19 pneumonia diagnosis and management. Our study aimed at assessing 1) CXR manifestations, frequency, and distribution; 2) the feasibility and repeatability of a CXR severity score; and 3) the correlation between the CXR severity score and clinical and laboratory parameters. We reviewed baseline CXRs and clinical data of consecutive patients who presented to our emergency department and resulted positive at SARS-CoV-2 reverse transcriptase–PCR oropharyngeal swab test from March 1, 2020 to April 6, 2020. Lung abnormalities and their distribution were analyzed. A score of CXR severity was assigned by two radiologists, independently, according to the extent of lung involvement, with a maximum score of 8 for CXR. Correlations between the CXR score and the clinical data were assessed. One hundred fifty-five patients were included; 143/155 (92%) were positive at baseline CXR. Ground-glass opacity was the most common finding (141/143, 99%). Involvement was mainly bilateral (96/143, 67%), with peripheral distribution (79/143, 55%). The mean CXR severity score was 3.3 (±2); interobserver agreement was excellent, with a Cohen’s *K* correlation coefficient of 0.901. The CXR score showed a significant positive correlation with C-reactive protein, lactate dehydrogenase, and fever duration, and a negative correlation with oxygen saturation. Chest X-ray findings are in line with those reported by computed tomography studies. The use of a visual CXR score, easy to assess and highly reproducible, can reflect the clinical severity and help the patients’ management.

## INTRODUCTION

From its outbreak in Wuhan, Hubei Province, China, COVID-19 spread worldwide, with 5 million confirmed cases, on May 22.^1^ Pneumonia represents its most common manifestation; therefore, chest computed tomography (CT) has taken on an important role, in the diagnosis, follow-up, and therapy efficacy evaluation of COVID-19 disease.^2–5^

Because of the constantly increasing numbers of positive patients, the execution of chest CT in all patients suspected or positive for COVID-19 infection is not feasible, because of the overwork in radiology departments and the need to designate a CT machine dedicated to COVID-19–suspected or positive patients only, with strict infection control protocols.^6^

The American College of Radiology^7^ recommends the use of chest CT only in selected hospitalized symptomatic patients and advises the use of a portable X-ray machine to avoid moving patients and to minimize the risk of cross infection.

Although chest X-ray (CXR) might have limited sensitivity for COVID-19 pneumonia, because it could miss subtle ground-glass opacifications (GGOs), it is important for the follow-up, evaluation of potential supervening complications, and the first-line evaluation of patients with a high pretest odds of COVID-19 pneumonia.^8^

Moreover, we have to consider that COVID-19 is rapidly spreading also in developing countries, where healthcare systems are weaker, and the availability of CT scanner is poor, and COVID-19 could potentially have the greatest impact.^9^

Thanks to its wide availability, quick execution, and acquisition at the patient’s bed, CXR represents a cheap first-line tool in the assessment of lung parenchyma abnormalities, also in COVID-19 patients. The appropriate use of CXR on arrival in the emergency department (ED) has been successful in speeding up the management of patients.^10^

The recent radiological literature has been focused on chest CT findings of COVID-19 pneumonia, whereas, to the best of our knowledge, only a few data are available on the radiographic appearance of this infection.^11–13^

Standardized quantitative reporting could be useful to define the disease severity and help clinical management. Therefore, our study aimed at assessing 1) the CXR manifestations of COVID-19 infection, their frequency, and distribution; 2) the repeatability of the CXR severity score from Wong et al.^11^; and 3) the correlation between the CXR severity score and clinical and laboratory parameters.

## MATERIALS AND METHODS

This retrospective study was approved by our Institutional Review Board; patients’ consent was obtained.

### Patients’ clinical and laboratory data.

We reviewed baseline CXRs, clinical data, and blood tests of consecutive patients admitted to our ED from March 1, 2020 to April 6, 2020, who complained symptoms suspected for COVID-19 infection (cough, fever, or dyspnea), with confirmed COVID-19 positivity reverse transcriptase–PCR (RT-PCR) test from the oropharyngeal swab, carried out in the first 5 days from admission.

Inclusion criteria were as follows: patients age > 18 years; RT-PCR testing positive result; and availability of complete clinical data and blood test analysis.

For each patient, the following data were collected by two radiology residents in consensus, under the supervision of an experienced radiologist, from our ED electronic database: symptoms on ED admission, oxygen saturation (SpO_2_), temperature (in Celsius degrees), days from the onset of fever, lactate dehydrogenase (LDH), C-reactive protein (CRP), and comorbidities (diabetes, arterial hypertension, chronic renal insufficiency, chronic obstructive pulmonary disease, asthma, cardiovascular disorders, and oncological history).

### Imaging acquisition and analysis.

All CXRs were acquired as computed or digital radiographs, in the posteroanterior or anteroposterior (AP) projection, based on the patients’ clinical condition, following our standard acquisition protocols.

**Figure 1.**
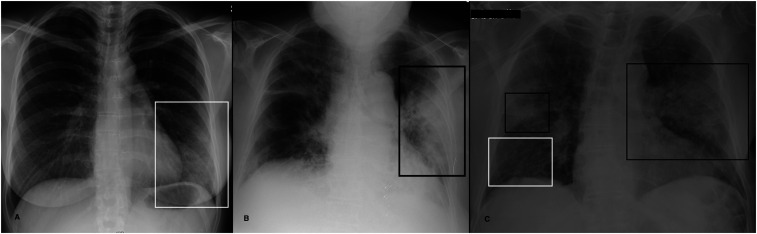
(**A**–**C**) Examples of chest X-ray abnormalities. (**A**) A 37-year-old woman with a 3-day fever, cough, and conjunctivitis. Chest X-ray shows a focal ground-glass opacity involving the lower field of the left lung (white rectangle). (**B**) An 86-year-old woman presented with dyspnea (SpO_2_ 88%), fever, and cough. Chest X-ray shows an area of consolidation in the middle-lower left fields (black rectangle). Ground-glass opacification (GGO) is recognizable in the right lung. (**C**) A 77-year-old man with a history of diabetes and arterial hypertension presented to the emergency department with a 7-day fever (38°C), dry cough, and dyspnea with low SpO_2_ (84%). Chest X-ray shows bilateral consolidations in middle lung fields (black rectangles). Bilateral areas of GGOs are also recognizable, particularly evident in the lower field of the right lung (white rectangle).

**Figure 2.**
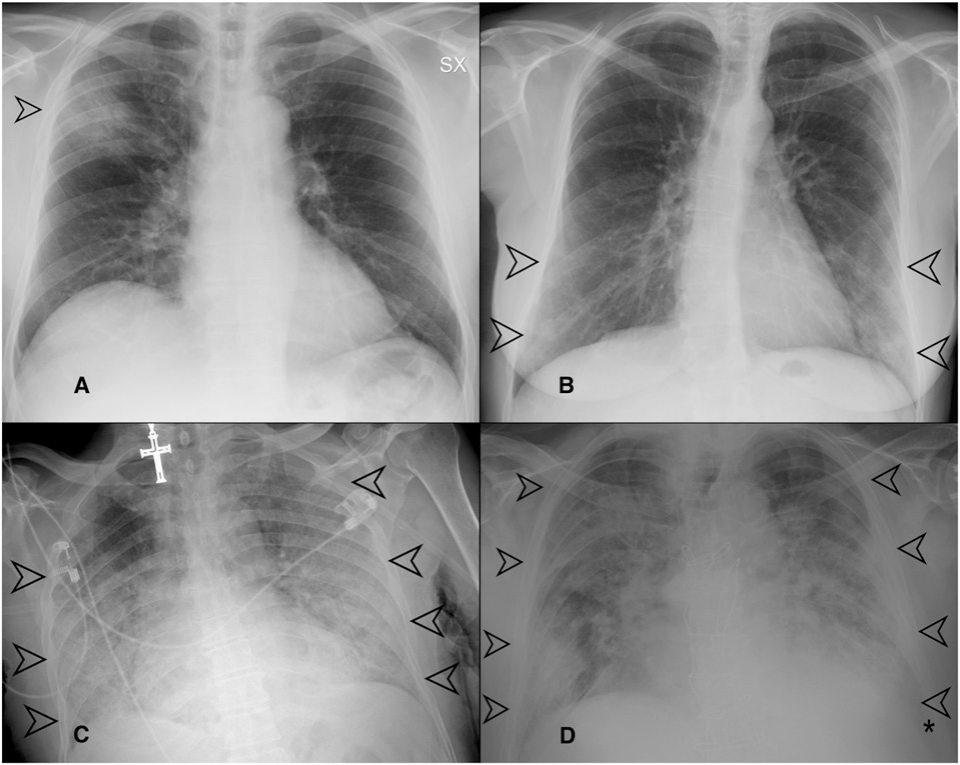
(**A–D**) Examples of chest X-ray (CXR) score assignment. (**A**) Chest X-ray showing a focal area of ground-glass opacifications (GGOs) in the upper field of the right lung (black arrow). The involvement of the right lung was < 25%; therefore, the CXR severity score assigned was 1. (**B**) Chest X-ray showing bilateral areas of GGOs involving the lower lung zones (black arrows). On both the left and right lungs, the involvement was < 50%; therefore, the score was 2 for each lung, with a global score of 4. (**C**) Chest X-ray showing huge areas of GGOs with bilateral involvement (black arrows), and saving of the upper field of the right lung; the extension on the left side was > 75% (score 4), whereas the involvement on the right side was < 75% (score 3); therefore, the overall score was 4 + 3 = 7. (**D**) Chest X-ray showing bilateral involvement, with areas of GGOs and consolidation (black arrows) involving all the lung fields. On both the left and right lungs, the involvement was > 75% (score 4); therefore, the global score was 4 + 4 = 8.

Two radiologists (MC, radiologist with 9 years of experience; MO, radiologist with 7 years of experience) in consensus assessed for each CXR: 1) the presence of lung abnormalities, diagnosed as consolidation, GGOs, or nodules, according to the Fleischner Society glossary of terms^14^ (Figure 1); 2) their distribution, classified into i) “peripheral” (the outer one-third of the lung), “central” (the inner two-thirds of the lung), or “both”; and into ii) “unilateral” or “bilateral.” The presence of pleural effusion was recorded.

A “CXR severity score,” according to Wong et al.,^11^ was assigned, independently, by two radiologists (MP, radiologist with 7 years of experience; GO, radiologist with 25 years of experience), depending on the extent of involvement by consolidation or GGOs (0 = no involvement, 1 = < 25%, 2 = 25–50%, 3 = 50–75%, and 4 = > 75% involvement), for each lung, with a maximum score of 8 for CXR. Some examples are provided in Figure 2.

### Statistical analysis.

Values were checked with a one-sample Kolmogorov–Smirnov test for normality. The interobserver agreement was calculated through the Cohen *k* coefficient. Correlations between values were evaluated through Spearman’s correlation coefficient. Kruskal–Wallis *H* test and Mann–Whitney *U* test were used to evaluate differences between independent groups. The relationships among the CXR severity score, radiological features, and clinical and laboratory parameters were investigated fitting a logistic regression model.

*P* < 0.05 was regarded as statistically significant. Statistical analysis was performed using SPSS 20 (IBM, Chicago, IL).

## RESULTS

### Patients’ clinical and laboratory data.

One hundred fifty-five patients (101, 65%, males, and 54, 35%, females; age range: 30–95 years; mean age: 64 ± 16 years) were included. Their clinical characteristics are summarized in Table 1*.* Fever (81%), cough (54%), and dyspnea (37%) were the most frequent symptoms. The average time from fever onset was 7 ± 3.6 days. The most common comorbidity was hypertension (38%).

**Table 1:** Characteristics of patients, clinical presentation, and comorbidities

Characteristic	Value
Number of patients (*N* = 155)	
Gender, *n* (%)	Male 101 (65)
	Female 54 (35)
Mean age (years), mean (±SD)	64 (±16.1)
Clinical presentation	
Fever, *n* (%)	126 (81)
High fever (T° > 38°C), *n* (%)	37 (24)
Days of fever, mean (±SD)	7 (±3.6)
Cough, *n* (%)	83 (54)
Dyspnea, *n* (%)	58 (37)
Gastrointestinal symptoms, *n* (%)	19 (12)
Chest pain, *n* (%)	8 (5)
Conjunctivitis, *n* (%)	3 (2)
Hemoptysis, *n* (%)	2 (1)
Oxygen saturation level (SaO_2_), mean (±SD) (%)	92 (±6.6)
Comorbidities, *n* (%)	
Hypertension	48 (31)
Cardiovascular disease	29 (19)
Diabetes	12 (8)
Chronic obstructive pulmonary disease	9 (6)
Oncological disease	9 (6)
Chronic kidney disease	6 (4)
Asthma	3 (2)

On admission, all patients underwent blood sampling and oropharyngeal swab test for RT-PCR analysis; negative tests in symptomatic patients were repeated up to three times. Baseline RT-PCR resulted positive in 143/155 (92%) of cases, with 12/155 (8%) falsely negative results.

### Image acquisition and analysis.

All 155 patients had CXR on admission; 125 (81%) were performed in the AP projection; 143/155 (92%) CXRs showed pulmonary abnormalities, whereas 12/155 (8%) were negative. Ground-glass opacification was the most common finding (141/143, 99%), followed by consolidation (60/143, 42%). Involvement was mainly bilateral (96/143, 67%), with prevalent peripheral distribution (79/143, 55%). Overall, radiographic findings are listed in Table 2.

**Table 2:** Chest X-ray acquisition, types of abnormalities, and their distribution

Characteristic	Value
CXR (*N* = 155), *n* (%)	
Positive CXR	143 (92)
Negative CXR	12 (8)
Anteroposterior acquisition, *n* (%)	125 (81)
Posteroanterior acquisition, *n* (%)	30 (19)
Chest abnormalities, *n* (%)	
GGOs	141 (91)
Consolidation	60 (39)
Both GGOs and consolidation	58 (37)
Pleural effusion	15 (10)
Distribution of parenchymal abnormalities, *n* (%)	
Peripheral	79 (51)
Central	20 (13)
Both	44 (28)
Bilateral	96 (62)
Monolateral	47 (30)

CXR = chest X-ray; GGOs = ground-glass opacifications.

The mean CXR score was 3.3 (±2); 61/155 (39%) of patients presented a low score (0–3), whereas 10/155 (6%) showed a very high score (7–8). Interobserver agreement in the CXR score assignment was excellent, with a Cohen’s *K* correlation coefficient of 0.901.

**Figure 3.**
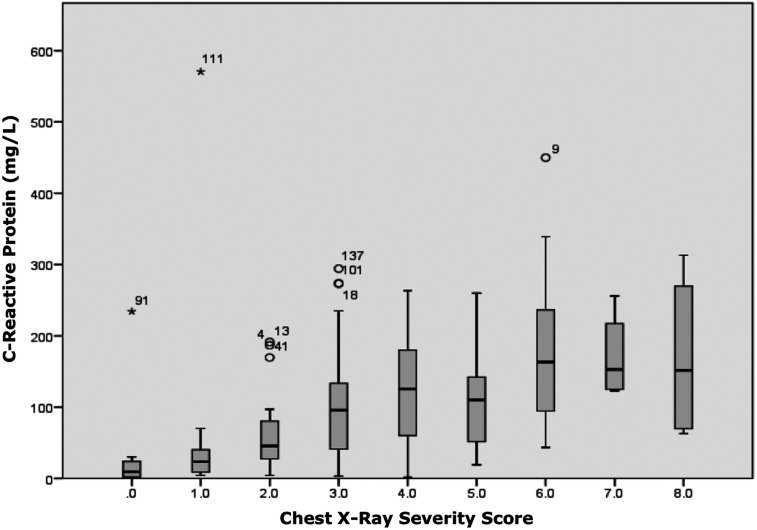
Chest X-ray severity score showed a significant positive correlation with C-reactive protein blood levels (*P* < 0.001; *r*-value 0.545). This figure appears in color at www.ajtmh.org.

**Figure 4.**
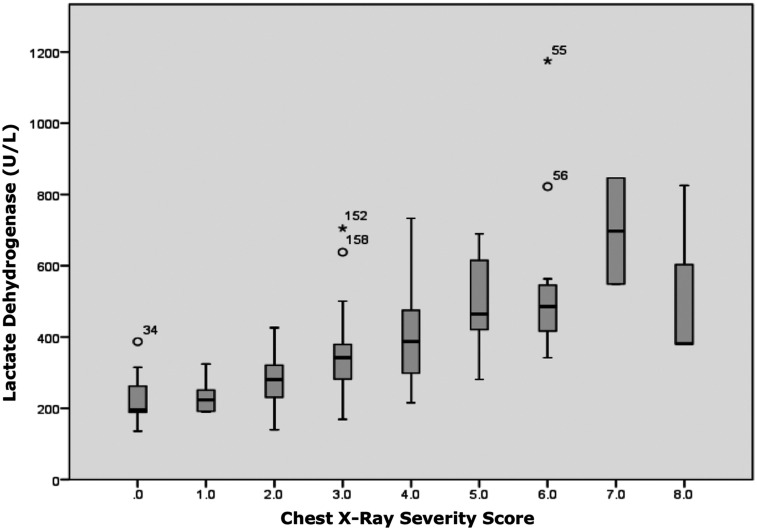
Chest X-ray severity score showed a significant positive correlation with lactate dehydrogenase blood levels (*P* < 0.001; *r*-value 0.770). This figure appears in color at www.ajtmh.org.

**Figure 5.**
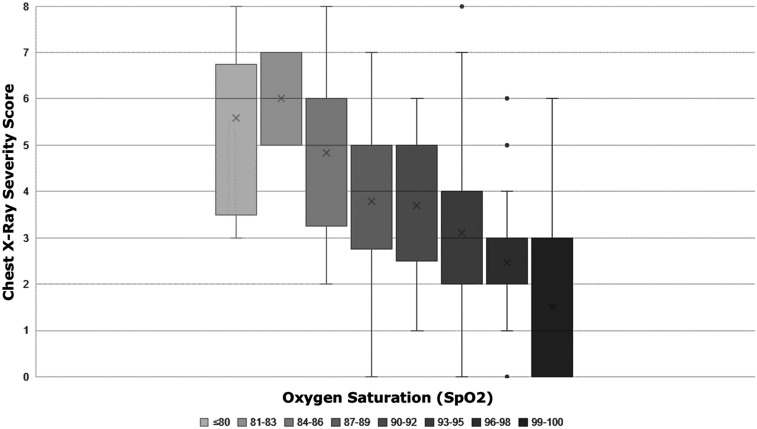
Chest X-ray severity score showed a significant negative correlation with oxygen saturation (*P* < 0.001; *r*-value −0.547).This figure appears in color at www.ajtmh.org.

The CXR score showed significant positive correlation with CRP (*P* < 0.001), LDH (*P* < 0.001), and fever duration (*P* = 0.01), and a significant negative correlation with SpO_2_ (*P* < 0.001), with *r*-values of 0.545, 0.770, 0.253, and −0.547, respectively (Figures 3–5). A very high CXR score (> 6) was found only in patients with dyspnea, at the limits of statistical significance (*P* = 0.06). No significant correlation was found between CXR score and temperature, cough, and comorbidities (*P* > 0.05).

## DISCUSSION

Chest X-ray is an essential tool for assessment of lung abnormalities in routine and emergency settings. Its main advantages are the wide availability, rapid execution, and acquisition at the patient’s bed with portable machines to limit the risk of cross infection.^6,10^

In the management of COVID-19–positive patients, it is important to define the severity of pneumonia.^15^ Chest X-ray is less sensitive than chest CT in mild or early COVID-19 pneumonia.^11^ However, the rapid spread of COVID-19 has resulted in scenarios characterized by high pretest probability, more advanced stages of the disease at presentation, and limited resources.^16^ As the prevalence of COVID-19 increases, CXR gains importance in diagnosis and definition of severity disease; therefore, it is important to validate a reporting method that allows not only the description of the disease but also the assessment of its grading in a quantitative and reproducible manner.

Wong et al.^11^ proposed a radiographic score for COVID-19 pneumonia based on the percentage extension of consolidation and GGOs. In their study, at baseline, the prevalent symptoms were fever (60%) and cough (41%); 31% of patients had normal CXR, and no patient had a CXR score > 6.

In our cohort, typical symptoms were more frequent (fever 81% and cough 54%); only 8% of patients presented negative CXR, and 6% of patients showed a high score (> 6). These differences can be explained by the fact that patients presented to our ED with a more advanced disease; therefore, in this context, CXR proved to be an adequate tool for COVID-19 pneumonia, detecting anomalies in the vast majority (92%) of positive cases on admission.

In the study by Wong et al.,^11^ consolidation was the most common finding (47%), followed by GGOs (33%). In our cohort, instead, GGOs were almost always present in positive CXRs (99%), accompanied by consolidation in 42% of cases, a result in line with findings previously described for CT.

COVID-19 pneumonia showed a characteristic distribution; both in our study and in the one by Wong et al., chest abnormalities were found predominantly bilateral in 67% and 63% of cases and with a peripheral distribution in 51% and 55% of CXR, respectively. These findings are in line with the features observed on chest CT.^2–5^

Quantifying COVID-19 pneumonia could be very important in the clinical management, and the tool must be as reproducible as possible. Our study analyzes the interobserver reliability of the CXR severity score, with an excellent result (Cohen’s *K* correlation coefficient = 0.901). Another study evaluated the agreement between radiologists, with a similar result.^17^

A significant direct correlation was found between the CXR severity score and blood levels of LDH and CRP. Moreover, the CXR score showed a significant inverse correlation, with SPO_2_ confirming that this score represents a good indicator of respiratory function. Direct correlation between COVID-19 pneumonia severity, blood values, and SPO_2_ has been previously reported for chest CT.^2,18,19^

Days from fever onset directly correlated with the CXR score. This is in agreement with the fact that the disease usually progresses in a few days from an almost asymptomatic phase to the development of severe pneumonia and that an early therapeutic approach to these patients could potentially reduce the critical cases.^20^ Chest X-ray score showed no correlation with comorbidities; for this reason, we can assume that the test is not altered by the presence of preexisting diseases, but the severity of pneumonia.

The results listed earlier need to be confirmed with a larger cohort of patients and performed in different hospitals and various conditions for validation. The main limitation of this study is the absence of a reference standard imaging for comparison, as chest CT was not routinely performed in COVID-19 patients at our institution.

In conclusion, our results support the role of CXR as a first-line diagnostic tool in symptomatic COVID-19 patients, in particular in a high pretest probability environment and/or limited resources scenario. Moreover, the use of a radiological score can result in a clearer communication with the clinicians and a more effective patient management.
